# Total hip arthroplasty following failure of tantalum rod implantation for osteonecrosis of the femoral head with 5- to 10-year follow-up

**DOI:** 10.1186/s12891-018-2219-z

**Published:** 2018-08-16

**Authors:** Qi Cheng, Jin-long Tang, Jiang-jiang Gu, Kai-jin Guo, Wang-shou Guo, Bai-liang Wang, Feng-chao Zhao

**Affiliations:** 1 Department of Orthopedic Surgery, The Affiliated Hospital of Xuzhou Medical University, No. 99 Huaihai West Road, Xuzhou, Jiangsu 221002 People’s Republic of China; 2 Department of Joint Surgery, China-Japan Friendship Hospital, Beijing, 100029 People’s Republic of China

**Keywords:** Core decompression, Trabecular metal implant, Conversion total hip arthroplasty, Hardware removal

## Abstract

**Background:**

Total hip arthroplasty (THA) with failure of tantalum rod implant for osteonecrosis of the femoral head (ONFH) will be the only choice for patients. However,it remains unknown whether tantalum rod implantation has an adverse effect on the survival time of implants following conversion to THA. The aim of this study was to retrospectively evaluate the clinical and radiographic outcomes of conversion to THA in patients who were previously treated with implantation of a tantalum rod.

**Methods:**

This study included 31 patients (39 hips), who underwent conversion to THA due to failure of core decompression with an implanted tantalum rod. Among these 31 patients, 26 patients were male and five patients were female. The mean age of these patients was 49.3 years old (range: 36–64 years old). The control group included 33 patients (40 hips), who underwent total hip replacement without tantalum rod implantation. The hip Harris score, implant wear, osteolysis, radiolucencies and surgical complications were recorded during the follow-up. The distribution of tantalum debris in the proximal, middle and distal periprosthetic femoral regions, radiolucent lines and osteolysis were analyzed on post-operative radiographs.

**Results:**

There were no significant differences in Harris score, liner wear and complications between the two groups (*P >* 0.05). Osteolysis and radiolucent lines more likely occurred in patients with tantalum debris distributed in three regions than in one or two regions (*P* < 0.05).

**Conclusions:**

The mid-term clinical outcome of patients who underwent THA with tantalum rod implantation was not different from those without a tantalum rod, suggesting that tantalum debris did not increase the liner wear rate. However, the distribution of periprosthetic tantalum debris in the proximal, middle and distal femoral regions may increase the risk of femoral osteolysis and radiolucent lines.

## Background

Osteonecrosis of the femoral head (ONFH) is a progressive disease due to decreased vascular supply to the subchondral bone of the femoral head, resulting in osteocyte death and collapse of the articular surface. This disease typically affects adults in the third to fifth decades of life, and incapacitates patients due to pain and decreased hip range of motion [1]. If the disease remains untreated at the early stage, approximately 70–80% of the patients will develop to secondary hip osteoarthritis, and total hip arthroplasty (THA) will be the only choice for patients with ONFH [2].

The primary goal during the early stages of ONFH is to preserve the hip joint, and several techniques have been implemented [3–5]. In recent years, core decompression combined with insertion of a porous tantalum rod has been developed for preventing and curing ONFH. Tantalum rods have a similar elastic modulus to the bone and the compressive strength of the cancellous bone, thereby providing good tissue compatibility. However, the clinical outcomes, postoperative weight-bearing time and role of porous tantalum implants remain controversial [2, 6, 7]. The failure of tantalum rods for ONFH has been reported in the last few years. The failure rates range from 2 to 56% [8–10]. Once the subchondral bone collapses, disease progression is difficult to reverse. Cracks to the femoral head, the narrowing of the joint space and the deterioration of joint function occur sequentially, and hip arthroplasty becomes the only remaining therapeutic option for these patients.

Approximately 5–18% of all hip arthroplasties are performed on patients with a primary diagnosis of osteonecrosis [11]. THA with failure of tantalum rod implants has recently been indicated in relatively young patients with a long lifespan, who may have a higher rate of revision surgeries than elderly patients. However, the mid-term follow up results of the implants have not been reported. In addition, it remains unknown whether tantalum rod implantation has an adverse effect on the survival time of implants following conversion to THA. Thus, we retrospectively evaluated the clinical and radiographic outcomes of the conversion to THA with a mid-term follow-up period of 5–10 years in patients previously treated with tantalum rod implantation. In addition, the clinical outcomes of these patients were compared with patients who underwent a similar THA without the insertion of a tantalum rod.

## Methods

### Patients

The Ethical Committee of our institution approved the present study. This retrospective study included 44 osteonecrotic hips in 35 consecutive patients, who were treated with THA following failure of core decompression with an implanted tantalum rod (Zimmer, USA) in the Affiliated Hospital of Xuzhou Medical University and China-Japan Friendship Hospital between June 10, 2007 and July 15, 2012. Patient data were collected retrospectively for more than 5 years. One patient (2 hips) died of diseases unrelated to the surgery and 3 patients (3 hips) were lost to follow-up. The 31 patients (39 hips; 5 women and 26 men) were available for review. The average age of these patients was 49.3 years old (range: 36–64 years old). The etiology of the osteonecrosis was idiopathic or unknown in 13 hips, and was due to the use of corticosteroids in six hips, alcohol abuse in 16 hips, and a traumatic event in four hips. The mean time between tantalum rod implantation and conversion to THA was 33.1 months (range: 16–63 months). Twenty-three hips in 20 patients were treated with ceramic-on-ceramic (CoC) implants, and 16 hips in 11 patients were treated with ceramic-on-polymer (CoP) implants. Anatomy stems (Ribbed, Link, Germany) were used in 14 hips and tapered stems (CLS, Zimmer, USA) were used in 25 hips. This study also included 40 hips in 33 patients with ONFH, and assigned this as the control group. Patients in the control group underwent primary THA for ONFH with the same type of implants during the same period.

### Surgical procedure

**Fig. 1:**
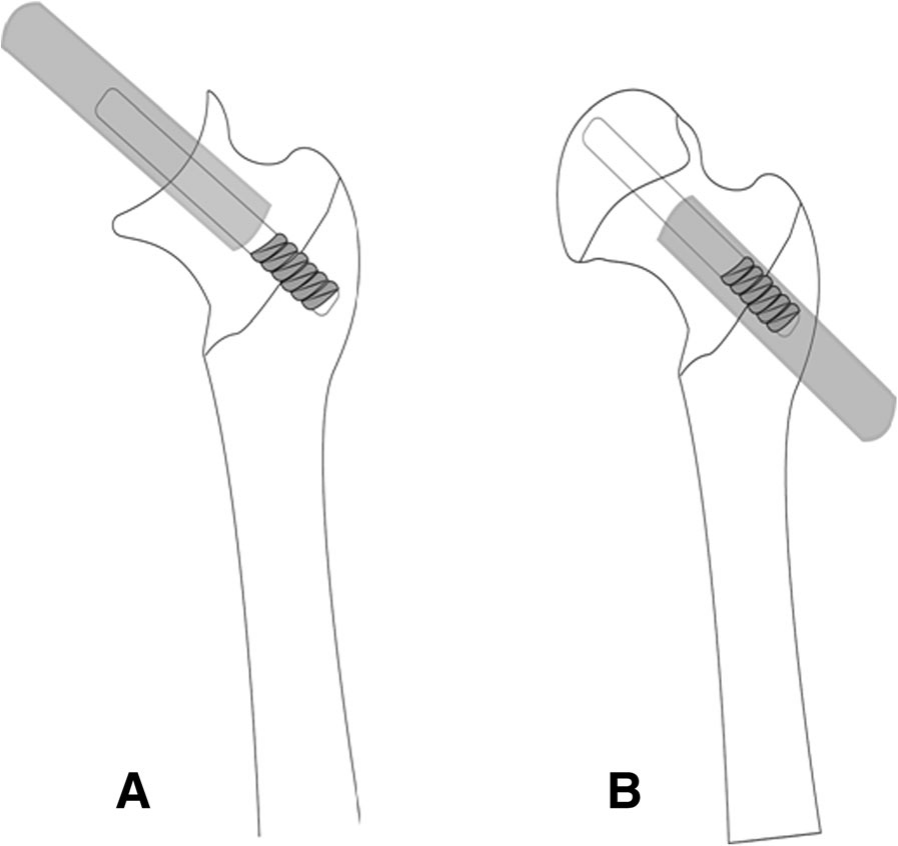
**a** The antegrade method. **b** The retrograde method

All patients were placed in the lateral decubitus position. For the standard posterolateral approach, a skin incision was made through the fascia over the greater trochanter, and the lateral cortical portion of the rods was exposed. The gluteus maximus was split, the external rotators were detached, and an incision was made on the hip capsule. The tantalum rod was removed through two methods. In the antegrade method, after exposing the femoral head and dislocating the hip, an oscillating saw blade was used to split the femoral neck. Based on the diameter of the implants, a medical metal-cutting trephine was chosen and placed over the proximal end of the tantalum rod. The rod was trephined from the level of the head to the lateral cortex of the femur in an antegrade direction. Then, the tantalum rods were removed without major bone loss. In the retrograde method, the rod was trephined from the lateral cortex of the femur to the level of the head in a retrograde direction, and the tantalum rods were removed without splitting the femoral neck. After the rod was removed, the tantalum particles imbedded in the bone were washed with normal saline. The lateral cortical hole was packed with a bone graft from the femoral head (Fig. 1).

For all patients, THA was performed using cementless femoral stems and an acetabular cup according to the standard surgical procedure. For patients with bilateral surgeries, the same implants were used. In the tantalum rod group, a 28-mm head with CoC implants was used in 11 hips, and a 32-mm head with CoC implants was used in 12 hips. In the control group, a 28-mm head with CoC implants was used in 10 hips and a 32-mm head with CoC implants was used in 15 hips. For CoP implants, a 28-mm head was used in seven hips and a 32-mm head was used in nine hips in the tantalum rod group. A 28-mm head was used in 10 hips and a 32-mm head was used in five hips in the control group.

### Clinical evaluation

**Fig. 2:**
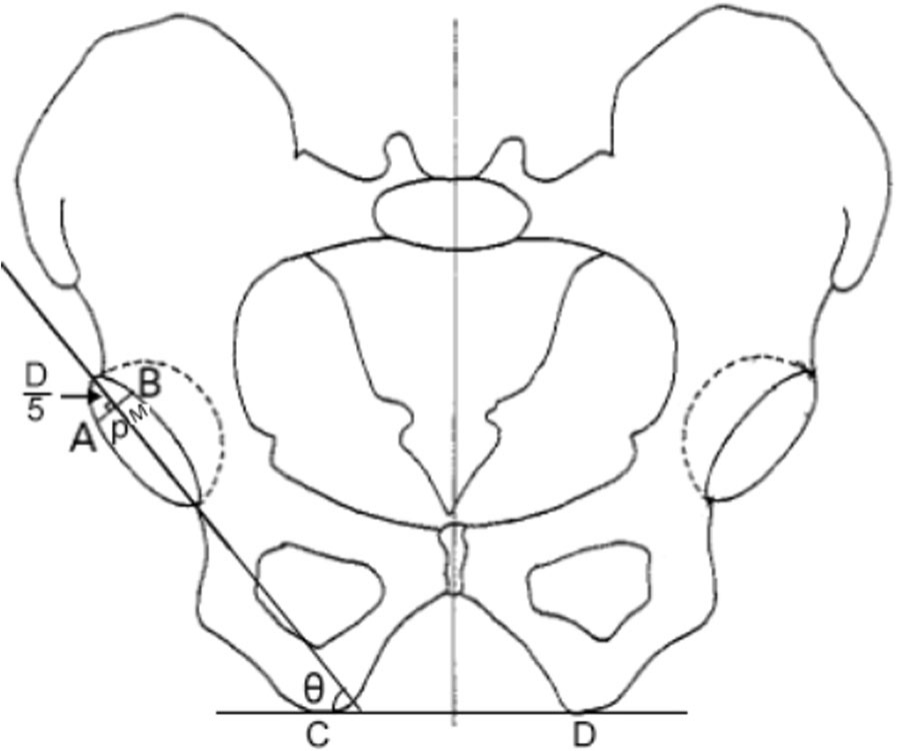
The point M is marked at one-fifth of the long diameter. Planar anteversion = arc of sin (p/0.8D). The inclination = θ

**Fig. 3:**
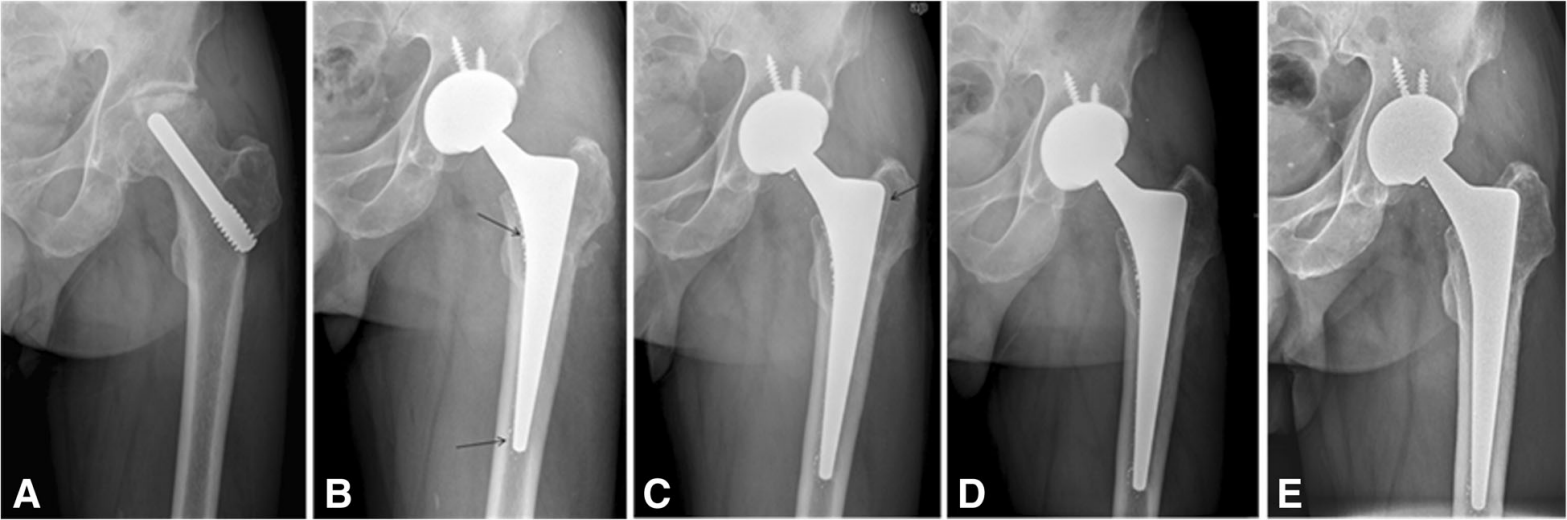
Radiographs of the left hip of a 46-year-old man with osteonecrosis of the femoral head (ONFH) after failure of the tantalum implant with conversion to total hip arthroplasty with ceramic-on-ceramic bearing. **a** Pre-operative radiograph. **b** Radiograph at 1-year, postoperatively, shows no osteolysis. Tantalum debris can be observed in the proximal, middle and distal regions (black arrows). **c** An anteroposterior view of the left hip at two years after total hip arthroplasty reveals a well-defined sclerotic edge near the medulla of the greater trochanter (black arrow). **d** Radiograph at four years, postoperatively, shows that the acetabular and femoral components are solidly fixed in a satisfactory position, with the radiolucencies in zones 1 and 7. **e** Radiograph at six years, postoperatively, shows no evidence of prosthesis loosening. The Harris score was 93 points at the last follow up

**Fig. 4:**
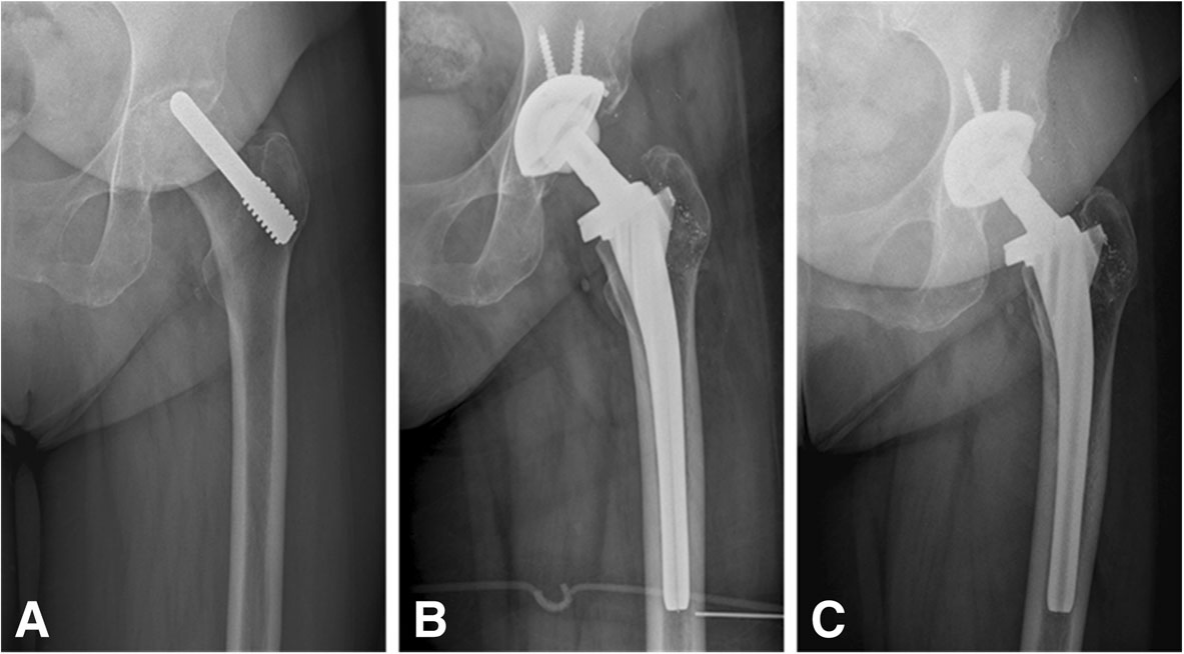
Radiographs of the left hip of a 51-year-old woman with ONFH after insertion of the tantalum implant with later conversion to total hip replacement with a ceramic-on-polyethylene bearing. **a** Pre-operative radiograph. **b** Anteroposterior postoperative radiograph at 1-year follow-up shows no osteolysis or radiolucencies. Tantalum debris can be observed in the femoral proximal region. **c** Radiograph at the 7-year follow-up shows no changes

All patients underwent clinical and radiographic examinations at one and three months, postoperatively, and at 6-month intervals thereafter. The clinical results were assessed using the Harris Hip Score (HHS) [12]. Thigh pain, complications and squeaking phenomenon, particularly those arising from CoC bearings, were also recorded. Anteroposterior (AP) pelvic and unilateral hip radiographs were routinely performed during the follow-up period. At 1-month follow-up, radiographs were evaluated to determine the inclination and anteversion of the acetabular component based on the AP film. A point M was marked at the 1/5th of the distance along the maximum diameter (D) of the projected ellipse of the wire marker. The AB line was a perpendicular line that passed through the M point and intersected the circle at A and B. The perpendicular distance (p) was measured from point A to point B. The formula of planar anteversion was calculated as follows: anteversion = arc of sin (p/0.8D). CD was a line that connected the ischial tuberosity at C and D. The nclination was the angle (θ) between the diametrical axis D and the line CD (Figs. 2, 3 and 4). For hips in the tantalum rod group, the distribution of tantalum debris was assessed at one week after surgery based on the AP unilateral hip radiographs. The femoral stem was divided into the proximal, middle and distal regions. The proximal region was defined by the region above the horizontal line of the lower point in the lesser trochanter. The middle and distal regions were separated equally between the lower point in the lesser trochanter and the tip of the stem. Femoral stem osteolysis and radiolucencies were classified, as previously described by Gruen et al [13] Furthermore, the acetabular components were evaluated according to the DeLee and Charnley classification of acetabular osteolysis and radiolucencies [14]. Periprosthetic osteolysis is defined as a circular or oval area of distinct bone loss with a diameter of > 2 mm [15]. A radiolucent line between the implant and the surrounding bone, usually parallel to the implant surface. Radiolucencies mostly remain constant and reflect a connective tissue layer. Bony demarcation of radiolucencies on the bone side, also known as sclerotic zones without irregularities or bone resorption. They are therefore considered to be a sign of absence of osseointegration. Subsequent osteolysis and radiolucencies has been recognized as the major cause of long term failure in total hip replacement. Acetabular liner wear was calculated using an Avenger Digital Caliper (Avenger Products, Boulder City, Nev) [16]. AP unilateral hip radiographs performed at one week postoperatively were used as a baseline, and wear measurements were performed on the most recent radiographs. The shortest distance between the acetabular cup interface and the center of the head of the prosthesis was measured, which was considered to be the point of maximum wear. Then, the initial thickness of the polyethylene cup was measured along the line of greatest wear. Measurements were corrected for magnification. The difference between two corrected values was calculated to determine the distance of the liner wear. The accurate value of this method is 0.075 mm. All X-Ray examinations were interpreted by one fellowship-trained academic musculoskeletal radiologist, who had 15 years of experience in interpreting hip XR examinations.

### Statistical analysis

All statistical analyses were performed using SPSS version 19.0 software (SPSS, Inc., Chicago, Illinois, USA). Demographic and clinical data were analyzed using Student *t*-test or Pearson’s chi-squared test between different groups. A *P*-value < 0.05 was considered statistically significant.

## Results

### Clinical results

Table 1 summarizes the clinical characteristics of patients in the tantalum rod group and control group. There were no significant differences in gender, age at time of surgery, BMI, primary diseases and the type of femoral stems between the two groups (*P* > 0.05, for five comparisons; Table 1).

**Table 1:** Clinical demographics of patients

Variable	Tantalum rod group		Control group	
	CoC	CoP	CoC	CoP
Patient (hip)	20 (23)	11 (16)	19 (25)	14 (15)
Gender				
Male	18	8	15	11
Female	2	3	4	3
Age (yr), mean (range)	41.4 (36–52)	46.2 (42–64)	43.2 (37–64)	45.6 (40–68)
Body mass index (kg/m^2^), mean (range)	25.2 (22.3–26.4)	24.7 (20.3–29.1)	24.6 (21.5–25.6)	23.2 (20.8–25.9)
Etiology				
Idiopathic	9	4	10	5
Corticosteroid	4	2	3	3
Alcohol	10	6	8	4
Trauma	0	4	4	3
Femoral Stem				
Anatomy stem	8	6	6	4
Tapered stem	15	10	19	11

*CoC* ceramic-on-ceramic, *CoP* ceramic-on-polymer

All patients were followed up for 7.4 years (range: 5–10 years). There were no differences in pre- or post-operative HHS values with CoC or CoP between the tantalum rod group and the control group (*P* > 0.05). Squeaking phenomenon was observed in five hips in the tantalum group and four hips in the control group. There were no significant differences in squeaking phenomenon between the groups (*P* > 0.05). No thigh pain, infections, dislocations, periprosthetic fractures and revision occurred during the follow-up period.

### Radiographic analysis

#### Ceramic-on-ceramic implants

There were no differences in acetabular inclination angle and acetabular anteversion angle between the tantalum rod group and the control group (*P* > 0.05). Acetabular periprosthetic osteolysis was not observed during the follow-up period. Femoral periprosthetic osteolysis or radiolucencies was observed in four hips in the tantalum rod group, but was not found in the control group (Fig. 1). The occurrence of femoral periprosthetic osteolysis or radiolucencies was significantly higher in the tantalum rod group than in the control group (*P <* 0.05). Osteolysis or radiolucencies was only involved in zones 1 and 7 in four hips (Fig. 1). The mean liner wear rate in the tantalum rod group was 20.3 um/yr. (range: 15.1–27.8 um/yr), which was not significantly different from that in the control group (mean: 18.9 um/yr.; range: 14.2–26.7 um/yr.; *P >* 0.05, Table 2).

**Table 2:** Outcomes and complications in the two groups with ceramic-on-ceramic

Variable	Tantalum rod group (*n* = 23)	Control group (*n* = 25)	*P*-value
Preoperative HHS	55.7 (49–63)	55.9 (34–68)	0.773
Postoperative HHS	95.1 (91–100)	94.2 (88–100)	0.202
Acetabular component			
Inclination	38.6 (32–44)	37.8 (33–45)	0.430
Anteversion	18.1 (13–25)	17.4 (10–27)	0.386
Femoral head (mm)			0.585
28	11	10	
32	12	15	
Squeaking phenomenon	5	4	0.719
Osteolysis or radiolucencies	4	0	0.022
liner wear rate (um/yr)	20.3 (15.1–27.8)	18.9 (14.2–26.7)	0.295

*HHS* Harris Hip Score

#### Ceramic-on-polymer implants

There were no differences in acetabular inclination angle and acetabular anteversion angle between the tantalum rod group and the control group (*P* > 0.05). Acetabular periprosthetic osteolysis or radiolucencies was not observed during the follow-up period. Femoral periprosthetic osteolysis or radiolucencies was observed in two hips in the tantalum rod group and one hip in the control group (Fig. 2). The osteolytic rate in the tantalum rod group was not significantly different from that in the control group (*P* > 0.05). Osteolysis or radiolucencies was involved in zones 1 and 7 in all three hips, and extended to zone 2 in one hip. The mean liner wear rate in the tantalum rod group was 68.9 μm/yr. (range: 34.5–89.6), which was not significantly different form that in the control group (mean: 56.5 μm/yr.; range: 23.5–102.7; *P >* 0.05, Table 3).

**Table 3:** Outcomes and complications in the two groups with ceramic-on-polymer

Variable	Tantalum rod group (*n* = 16)	Control group (*n* = 15)	*P*-value
Preoperative HHS	54.4 (38–63)	55.8 (47–65)	0.949
Postoperative HHS	95.3 (88–100)	94.3 (83–98)	0.328
Acetabular component (°)			
Inclination	37.2 (31–43)	38.4 (32–48)	0.347
Anteversion	19.2 (14–28)	17.6 (11–26)	0.163
Femoral head (mm)			0.200
28	7	10	
32	9	5	
Osteolysis or radiolucencies	2	1	1.000
liner wear rate (μm/yr)	68.9 (34.5–89.6)	56.5 (23.5–102.7)	0.155

*HHS* Harris Hip Score

#### The analysis of osteolysis or radiolucencies in the different tantalum debris groups

Tantalum debris was mainly located in the femoral region. For all hips in the tantalum rod group, tantalum debris in the proximal region was found. Moreover, tantalum debris was also present in the middle region in 11 hips and in the whole regions in 14 hips. For methods used to remove the tantalum rod, the debris distribution rates were significantly lower in the antegrade method (44.4%, 8/18) than in the retrograde method (81.0%, 17/21) (*P <* 0.05). The incidence rate of osteolysis or radiolucencies at the last follow-up time was 7.1% (1/14) in the proximal region, 0% (0/11) in the proximal and middle region, and 35.7% (5/14) in the whole region. Osteolysis or radiolucencies more likely occurred in the whole region (*P <* 0.05). However, there were no differences in the occurrence of osteolysis or radiolucencies between the other two groups without the distal region (*P >* 0.05, Table 4).

**Table 4:** Characteristics of six patients with osteolysis in the tantalum rod group

Case	Gender	Age (years)	prosthesis	Femoral head (mm)	Tantalum debris	Follow-up (years)	Osteolytic or radiolucency zones	HHS (points)
1	Male	46	CoC	28	Proximal, middle and distal	6	1,7	93
2	Female	51	CoP	28	Proximal, middle and distal	8	1,7	92
3	Male	57	CoP	32	Proximal, middle and distal	9	1, 2 and 7	93
4	Male	52	CoC	32	Proximal, middle and distal	6	1,7	90
5	Male	48	CoC	28	Proximal	8	1,7	95
6	Female	50	CoC	28	Proximal, middle and distal	6	1,7	91

*CoC* ceramic-on- ceramic, *CoP* ceramic-on-polymer, *HHS* Harris Hip Score

## Discussion

ONFH typically affects young patients in their one third to fifth decades of life, and a considerable proportion of patients suffer from bilateral hip involvement. Core decompression and tantalum rod implantation is a commonly used prophylactic surgery for the treatment of ONFH in pre-collapse osteonecrosis (prior to Ficat and ARCO stage II, and Steinberg stage III). However, the clinical outcomes and role of porous tantalum implants in preventing necrosis progression remains controversial [17, 18]. Although core decompression for Steinberg stage I and Steinberg stage II disease was successful as a definitive procedure in > 80% and 60% of patients, respectively, approximately 30–71% of treatments fails and converts to THA [11, 18]. The conversion to THA is technically demanding in terms of removing the metallic rod, increased blood loss, prolonged operative time, bone loss and the potential risk of femoral fracture [19, 20]. Olsen et al. [6] compared the clinical outcome of 21 patients (21 hips), who received THA following a failed index procedure for tantalum rod implantation, with a cohort of 21 patients (21 hips), who received primary THA for the same diagnosis. They found that there were no significant differences in the survival, clinical, or radiographic outcomes between two groups during a mean follow-up period of 24 months. However, the mid-term clinical outcome of THA following failure of tantalum rod implantation for the treatment of ONFH remains unclear. In the present study, the mid-term outcomes of conversion to THA in patients previously treated with tantalum rod implantation were investigated. It was found that the mid-term clinical outcomes of THA with tantalum rod implantation were accepted, and the tantalum debris did not increase the liner wear rate. However, the distribution of periprosthetic tantalum debris in the whole region of the femoral stem may increase the risk of femoral osteolysis and radiolucencies.

It was also found that there was no difference in the liner wear rate of CoC bearings between the tantalum rod group and control group at the > 5 year follow-up. The wear rate (20.3 um/yr. in the tantalum rod group and 18.9 μm/yr. in the control group) of implants with ceramic bearings is similar to that reported in literature. For example, Lewis et al. [21] reported a wear rate of 20 μm/year for ceramic bearing and 100% survivorship at a mean follow-up period of 10.9 years (56 hips/55 patients). Amanatullah et al. [22] reported that the mean liner rate was 30.5 μm/year in ceramic-ceramic implants (125 hips) and 94.4% survivorship (196 hips) at a mean follow-up of five years. For high cross-linked polyethylene, the liner wear rates range from 37 μm/year to 60 μm/year during a follow-up period of at least 10 years [23–25]. The porous tantalum debris, which is produced by reaming, is mainly visible in the femoral canal. Therefore, tantalum debris may not potentially cause third-body wear, and no correlation between the existence of tantalum debris and liner wear rate was found.

The osteolytic rate was 17.4% in the present ceramic cohort in the tantalum rod group, which was obviously higher than that in literature. For example, D’Antonio et al. [26] reported that osteolysis or radiolucencies occurred in 26% of metal-on- polyethylene patients (72 hips), but this did not occur in patients with alumina bearings (144 hips). Lau et al. [27] reported that there were no cases with acetabular or femoral osteolysis in 126 hips with third generation ceramic bearings during a > 10-year follow-up. Furthermore, it was found that the osteolytic rate was 12.5% in the present CoP group with tantalum rod implantation, which was similar to that (6.7%) in the control group. Furthermore, it has been reported that acetabular polyethylene cups with annual wear rates of under 80 mm^3^, between 80 and 140 mm^3^, and more than 140 mm^3^ were associated with very little, low-to-moderate, and high levels of osteolysis [28]. Dowd et al. [29] found that polyethylene wear rates of < 0.1 mm/y were not associated with osteolysis or radiolucencies, but wear rates higher than 0.3 mm/y were associated with 100% incidence of osteolysis in a 10-year follow up study. In the present study, the wear rate in the tantalum rod group with a CoP implant was very low (68.94 um/y). Thus, its adverse effect was limited. The porous tantalum debris retained in the cavity could be tolerated by the liner, but this did not cause third-body wear. However, radiographic findings revealed that there was a significant correlation between the distribution range of tantalum debris and femoral osteolysis or radiolucencies, and in CoC or CoP implants, especially for tantalum debris that extend to all regions around prosthesis. Olsen et al. [6] reported that a high rate of retained tantalum debris on post-operative radiographs may be a risk of accelerated time of THA revision. Tantalum particles at high concentrations may cause an inflammatory reaction and disrupt the balance between bone tissue formation and break down [30, 31]. Furthermore, the retained debris at the interface of the cementless prosthesis and femur can cause mechanical instability, which is a risk factor of periprosthetic osteolysis and aseptic loosening [32].

Despite the substantial improvements in cementless THA implants, periprosthetic osteolysis and subsequent aseptic loosening can not be avoided as time goes by. Osteolysis usually leads to implant loosening and the need for revision arthroplasty, which is associated with poorer clinical outcome and shorter survival, compared with primary THA [33]. Following the failure of tantalum rod implantation, many younger people with ONFH have to choose THA to complete pain-free activities of daily living. Thus, it is important to improve the survival time of primary THA and postpone the time of revision in patients with ONFH. A suitable trephine should be used to remove a trabecular metal screw [19], and a high-speed burr should be used to clear the joint space, in order to decrease the amount of tantalum debris retained in the periprosthetic cavity. Furthermore, in order to decrease the debris, the rod should be trephined from the level of the head to the lateral cortex of the femur in an antegrade direction.

The present study has some limitations. First, the study included patients with ONFH from many etiologies such as alcoholism, steroid use and hip trauma. It remains unclear whether different etiologies affect osteolysis or radiolucencies. Second, it remains difficult for plain radiology to detect pelvic and femur osteolysis. Therefore, the incidence of osteolysis and radiolucencies in the present study may be less than that evaluated by computed tomography (CT) [34]. Third, this study only measured femoral head penetration, but volumetric wear was not measured.

## Conclusion

In conclusion, it was found that the mid-term clinical outcomes of THA following failure in the tantalum rod group were good in patients with ONFH, when compared with THA without tantalum rods. The tantalum debris did not increase the liner wear rate. However, it should be noted that the distribution of periprosthetic tantalum debris in whole femoral stem regions may be associated with osteolysis at mid-term follow-ups.

## Abbreviations

AP: Anteroposterior
CoC: Ceramic-on-ceramic
CoP: Ceramic-on-polymer
CT: Computed tomography
HHS: The Harris Hip Score
ONFH: Osteonecrosis of the femoral head
THA: Total hip arthroplasty
